# Effects of caffeine gum on same-day and subsequent neuromuscular performance under a standardized resistance-priming condition in male basketball players

**DOI:** 10.1080/15502783.2026.2682313

**Published:** 2026-06-05

**Authors:** Jingmiao Wang, Bingxue Li, Yimin Huang, Yanhao Tu, Haolin Yang, Yunmei Chai

**Affiliations:** a School of Sport Training, Chengdu Sport University, Chengdu, Sichuan, People's Republic of China; b School of Physical Education, Chengdu Sport University, Chengdu, Sichuan, People's Republic of China

**Keywords:** Caffeine gum, resistance priming, neuromuscular performance, basketball players, subsequent performance

## Abstract

**Purpose:**

This study examined whether 3 mg·kg^−1^ (CAF3) and 6 mg·kg^−1^ (CAF6) caffeine gum, administered before a standardized resistance-priming (RP) protocol, produced additional neuromuscular performance advantages over placebo (PLA) at 1 h, 24 h, and 48 h in male basketball players.

**Methods:**

Fifteen male basketball players completed three randomized, double-blind, placebo-controlled crossover conditions: PLA+RP, CAF3+RP, and CAF6+RP. After 15 min of gum chewing, participants performed a standardized warm-up followed by RP consisting of 3 × 3 back squats at 85% 1RM. Mean concentric velocity (MV) was recorded during RP. Performance tests were conducted at 1 h, 24 h, and 48 h post-RP and included isometric mid-thigh pull peak force and 200-ms rate of force development, countermovement jump height, 20-m sprint, Y-shaped reactive agility, and Lane Agility Test. Data were analyzed using linear mixed-effects models adjusted for experimental period and administration sequence.

**Results:**

Significant dose × time interactions were observed for all performance outcomes except the Lane Agility Test. At 1 h, CAF3+RP and CAF6+RP produced superior performance compared with PLA+RP in isometric strength, explosive power, sprint, and reactive agility outcomes. No significant differences were observed between CAF3 and CAF6. At 24 h and 48 h, no additional between-condition advantages were found for either caffeine dose compared with PLA. Barbell MV during RP was higher under caffeine conditions than PLA, indicating greater RP training output.

**Conclusion:**

Within a standardized RP context, CAF3 and CAF6 caffeine gum were associated with improved same-day neuromuscular performance and higher RP barbell velocity compared with PLA, but no additional between-condition advantages were observed at 24 h or 48 h. Because this study did not include pre-intervention baseline testing, a caffeine-only condition, or a no-RP control condition, the findings should be interpreted as caffeine-related differences within an RP context rather than direct evidence of the independent effects of caffeine, RP, or their interaction.

## Graphical abstract

**Figure:**
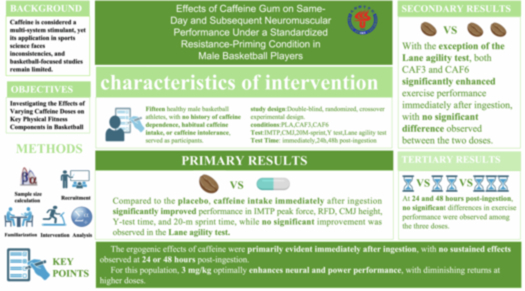

## 1. Introduction

Caffeine is one of the most widely used and well-supported ergogenic aids in sport [1]. As an adenosine receptor antagonist, it reduces the perception of fatigue by the brain and enhances central nervous system excitability and motor unit recruitment [2]. Acute caffeine ingestion has been shown to improve various aspects of neuromuscular performance [3]. Its ergogenic effects are influenced by both dosage and mode of administration. Doses of 3 mg·kg^−1^ (CAF3) and 6 mg·kg^−1^ (CAF6) are commonly reported to enhance exercise performance [4]. However, higher doses do not necessarily confer additional benefits and may increase the risk of adverse effects [5], indicating that the optimal caffeine dose remains inconclusive. Among different delivery forms, caffeine gum has recently gained attention due to its rapid onset. Caffeine released during chewing can be quickly absorbed through the highly vascularises buccal mucosa [6]. Compared with capsules, caffeine gum offers faster absorption and earlier ergogenic effects [7], making it particularly suitable for sports with limited pre-competition preparation time, such as basketball. Basketball competitions are often characterised by congested schedules (e.g. back-to-back games). Therefore, it is practically relevant to examine strategies that may enhance same-day performance and determine whether any additional performance advantages remain evident over the subsequent 24–48 h. Resistance priming (RP), a pre-competition training strategy, has received increasing attention due to its practical applicability. RP involves performing high-intensity resistance exercise 1–48 h before competition, aiming to enhance subsequent performance through the delayed potentiation effect (DP) [8]. However, whether different doses of caffeine gum produce additional neuromuscular performance benefits over placebo at 1 h, 24 h, and 48 h when administered before a standardised RP protocol in basketball players remains unclear.

Within RP protocols, the back squat is one of the most commonly used exercise modalities, and appropriately designed RP protocols may influence neuromuscular performance within a 1–48 h window [9–11]. However, subsequent performance responses following RP may depend on the balance between potentiation-related effects and fatigue [12]. Specifically, delayed performance enhancement is more likely to be observed when fatigue has dissipated while potentiation-related effects remain present [13]. Therefore, strategies that may help control fatigue and enhance neuromuscular activation during the RP stimulus could potentially improve training output and subsequent performance responses within an RP context, with caffeine representing a possible adjunct strategy. Alonso et al [14]. reported that CAF3 significantly increased barbell velocity and power output during squats performed at 50–90% 1RM, with greater benefits observed at higher loads. Similarly, Berjisian et al [15]. found that CAF6 significantly improved squat force output. These findings suggest that caffeine may enhance squat training output and may be associated with improved same-day performance within an RP context. However, direct evidence remains limited regarding whether different doses of caffeine gum, when administered before a standardised RP protocol, produce superior neuromuscular performance compared with placebo during the same-day and subsequent 24–48 h period.

Taken together, based on the available evidence, the present study aimed to determine whether CAF3 and CAF6, when administered before a standardised RP protocol, would produce caffeine-related neuromuscular performance advantages over placebo (PLA) at 1 h, 24 h, and 48 h. Importantly, because the present design compared CAF+RP with PLA+RP and did not include trial-day pre-intervention baseline testing, a caffeine-only condition, or a no-RP control condition, this study was able to evaluate caffeine-related between-condition differences within a standardised RP context, but was not designed to directly determine the independent effects of caffeine, the independent effects of RP, or whether caffeine augments or prolongs RP-induced delayed potentiation. The present study hypothesised that, compared with PLA+RP, CAF3+RP and CAF6+RP would be associated with higher RP training quality and superior performance at 1 h post-RP, whereas the presence of additional between-condition advantages at 24 h and 48 h was considered exploratory. We further hypothesised that CAF3 would produce effects comparable to those of CAF6.

## 2. Methods

### 2.1. Participants

A priori sample size was estimated using G*Power 3.1. For repeated-measures ANOVA within factors, with an effect size of f = 0.25, *α* = 0.05, and power = 0.80, a minimum of 15 participants was required. Allowing for a 10% dropout rate, 17 healthy male basketball players from Chengdu Sport University were recruited. Participants were eligible if they were healthy, free from lower-limb musculoskeletal injury or cardiovascular disease, low habitual caffeine consumers (<100 mg·day^−1^ or < 3 mg·kg^−1^·day^−1^), and had no history of caffeine dependence, withdrawal symptoms, intolerance, or allergy. They were also required to avoid strenuous exercise, caffeine, and alcohol for 24 h before testing. All participants provided written informed consent. Two participants withdrew because of injury during the intervention period; therefore, 15 participants were included in the final analysis. The study was approved by the Ethics Committee of Chengdu Sport University (approval number: 202675) (Table 1).

**Table 1.** Participant characteristics.

Height(cm)	Weight(kg)	Age(years)	Training Experience(years)
185.1 ± 3.67	75.9 ± 7.84	19.0 ± 0.94	7.5 ± 1.35

### 2.2. Experimental design

**Figure 1.**
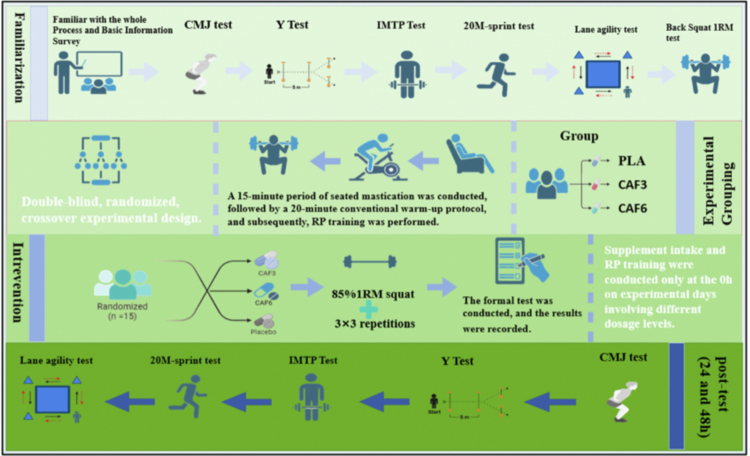
Experimental design overview. The figure summarises the familiarisation procedures, randomised crossover grouping, caffeine gum or placebo intervention, standardised resistance-priming protocol, and post-intervention testing schedule at 1 h, 24 h, and 48 h.

This study employed a randomised, double-blind, PLA-controlled crossover design. Trials were separated by a washout period of at least 5 days to reduce potential residual effects [16]. During familiarisation, participants’ anthropometric data were recorded for individualised caffeine dosing, and they practiced all performance tests. Back squat *One Repetition Maximum* (1RM) was also assessed to prescribe the RP load. For each experimental condition, the Day 1 procedure began at 10:00 a.m., defined as the start of gum supplementation and the RP protocol sequence. Randomisation was performed by a researcher not involved in data collection or analysis using opaque sealed envelopes, ensuring that participants and field testers were blinded to the assigned condition. After confirming compliance with pre-trial restrictions, including avoidance of strenuous exercise and caffeine intake for 24 h, participants chewed the assigned gum for 15 min at a self-selected chewing rate under natural chewing and swallowing conditions. Chewing frequency was not standardised or directly monitored, and participants were instructed to chew the gum in their usual manner throughout the 15-min period. Natural swallowing of saliva was permitted, and the gum was discarded immediately after chewing. Participants then completed a 20-min standardised warm-up, an approximately 20-min RP protocol, and 60 min of passive recovery. Accordingly, the same-day performance tests were conducted at approximately 11:40 a.m., corresponding to 1 h post-RP. To minimise differences in testing time, the 24 h and 48 h follow-up tests were scheduled at a similar clock time. Specifically, participants arrived at approximately 11:00 a.m. and performed the testing battery at approximately 11:40 a.m. All performance tests were completed in a fixed order. Supplementation and RP were performed only on Day 1 of each condition. Experimental procedure is in Figure 1.

#### 2.2.1. Resistance priming protocol

Participants performed a standardised RP protocol consisting of 3 sets of 3 repetitions of the back squat at 85% 1RM, with 3 min of rest between sets. The design of this RP protocol was primarily based on the balance between potentiation and fatigue. A relatively high squat load may facilitate the recruitment of high-threshold motor units, whereas the low-volume structure may reduce the accumulation of residual fatigue and help maintain movement quality [17]. In addition, given that basketball performance is highly dependent on lower-limb explosive power [18], the back squat may represent a sport-relevant RP exercise [19].

During the exercise, Participants were instructed to perform each squat with a controlled, self-selected eccentric descent using their normal squat technique, avoiding a rapid or ballistic lowering phase, followed by an explosive concentric ascent. Previous research has suggested that movement velocity can serve as an objective indicator of training quality [20]. Therefore, mean concentric velocity (MV) was extracted for each repetition [21], and the set-level MV reported in the present study represented the average MV of the three repetitions within that set. To monitor within-set velocity decline, velocity loss within each set was calculated relative to the MV of the first repetition in that set, with a 10% velocity-loss threshold used as the monitoring criterion [22].

#### 2.2.2. Dietary recording and pre-trial readiness control procedures

At the first laboratory visit, participants were provided with a food diary by the research staff and received standardised instructions. They were asked to record their dietary intake during the 48 h before each experimental condition, as well as their habitual caffeine intake, to verify that they met the criteria for low habitual caffeine consumption (<100 mg·day^−1^ or < 3 mg·kg^−1^·day^−1^) [23,24]. To minimise fluctuations in trial-day baseline readiness, participants were instructed to avoid strenuous exercise and caffeine intake for 24 h before each formal trial and to maintain relatively consistent sleep and daily activity patterns before testing. All formal experimental trials for each participant were conducted at a similar time of day, with testing sessions scheduled within ±1 h of the participant’s first trial start time, to minimise the potential influence of circadian variation. However, no pre-intervention performance baseline was collected on each experimental trial day. Therefore, it was not possible to directly confirm whether participants’ immediate physiological readiness was comparable before each condition. Recent evidence indicates that even a single night of sleep extension can improve morning physical and cognitive performance, suggesting that acute changes in sleep status may influence morning readiness and performance output [25]. Although the present study attempted to reduce day-to-day readiness fluctuations through standardised pre-trial behavioural requirements, their potential influence on the 1 h results cannot be completely excluded.

### 2.3. Testing procedures

#### 2.3.1. 1RM back squat test

Back squat 1RM was used to prescribe the absolute RP load. Previous research supports the reliability of RM- and 1RM-related strength testing in male athletes [26].

After 5 min of low-intensity cycling and standardised dynamic stretching, participants completed progressive squat-specific warm-up sets at approximately 50%, 70%, and 85–90% of the estimated 1RM. The load was then adjusted by 5–10% after a successful attempt or reduced by 2.5–5% after a failed attempt until 1RM was determined within 3–5 maximal attempts, with 3–5 min of rest between attempts. All tests were performed in a power rack, and a valid repetition required the thighs to reach at least parallel to the floor.

#### 2.3.2. Countermovement jump (CMJ)

CMJ height was assessed using a three-dimensional force platform (Kistler 9286AA, Kistler, Switzerland). CMJ height was selected as a practical indicator of lower-limb dynamic explosive power, as multi-joint lower-limb strength parameters are closely related to vertical jump performance [27]. Participants performed the jump with hands on hips, using a self-selected countermovement depth, and were instructed to jump vertically as high as possible while avoiding lateral rotation. After three practice jumps, each participant completed two formal trials separated by 60 s of rest. The best jump height was retained for analysis.

#### 2.3.3. 20-m sprint

The 20-m sprint test was performed using a Smart Speed timing system (Fusion Sport, Australia). Timing gates were positioned at 0 m and 20 m, with both gates set at a height of 0.75 m. Participants started 30 cm behind the first timing gate, and timing was triggered automatically when they crossed the start gate. After one practice trial, participants completed two formal trials separated by 120 s of rest. The fastest 20-m sprint time was retained for analysis.

#### 2.3.4. Isometric mid-thigh pull (IMTP)

The IMTP test was performed on a three-dimensional force platform (Kistler 9286AA, Kistler, Switzerland) sampling at 1000 Hz, with the vertical force range set at 20 kN [28]. Bar height was adjusted individually so that the pulling posture resembled the second-pull phase of weightlifting, with the trunk nearly upright and the knee angle controlled between 120° and 130° [29]. Participants used lifting straps to minimise the influence of grip strength and were instructed to pull “as fast and as hard as possible” until the stop command. Each participant completed two trials separated by 120 s of rest. Peak force and 200-ms RFD were automatically calculated by the software, and the best trial was retained for analysis.

#### 2.3.5. Y-shaped reactive agility test (Y Test)

The Y-shaped reactive agility test was used to assess reactive agility. The course consisted of a 5-m central stem and two 3-m branch arms positioned at approximately 45°. Participants sprinted forward from the starting line and, after crossing the second line, responded to a randomly illuminated left or right directional signal by sprinting to the corresponding finish gate. A 1-s delay was introduced before the directional signal to reduce anticipatory movement. Each participant completed two trials separated by 60 s of rest. The fastest completion time was retained for analysis.

#### 2.3.6. Lane agility test

The Lane Agility Test was used to assess basketball-specific agility and multidirectional movement ability, including rapid change of direction, body control, and movement efficiency [30]. Participants completed two consecutive mirrored movement sequences at maximal effort while keeping the torso facing the baseline throughout the test. Each participant completed two trials separated by 120 s of rest. The fastest completion time was retained for analysis.

### 2.4. Statistical analyses

All statistical analyses were performed using SPSS 27.0, with data presented as mean ± SD. Linear mixed-effects models (LMMs) were utilised for the primary analyses. For all models, the normality assumption was assessed by inspecting the Q-Q plots of the model residuals. For performance outcomes, dose, time, dose × time, experimental period, and administration sequence were included as fixed effects, with participant as a random intercept. Significant interactions were followed by Bonferroni-adjusted pairwise comparisons based on estimated marginal means, reported as MD, SE, 95% CI, and adjusted *p* values. Hedges’ g with small-sample correction was calculated for key CAF versus PLA comparisons at 1 h. MV during RP was analysed using a separate LMM with dose, set, dose × set, period, and sequence as fixed effects. Statistical significance was set at *p* < 0.05.

## 3. Results

**Figure 2.**
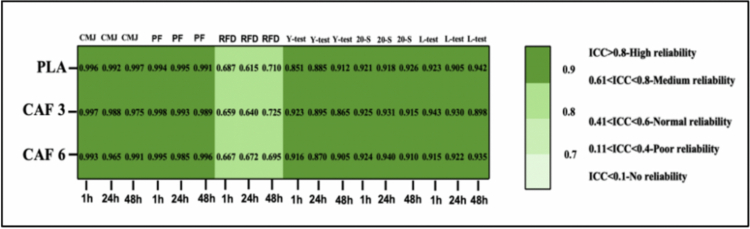
Test reliability. Intraclass correlation coefficients are shown for each performance test across placebo, CAF3, and CAF6 conditions at 1 h, 24 h, and 48 h. CMJ, IMTP peak force, Y-shaped reactive agility, 20-m sprint, and Lane Agility Test showed good to high reliability, whereas IMTP 200-ms RFD showed moderate reliability.

To assess test reliability, ICCs were calculated for each performance test across intervention conditions using a two-way mixed-effects model, absolute-agreement definition, and single-measure form. ICCs exceeded 0.75 for CMJ, IMTP peak force, Y-shaped reactive agility, Lane Agility Test, and 20-m sprint, indicating good reliability, whereas IMTP 200-ms RFD showed moderate reliability with ICCs > 0.60 (Figure 2).

**Figure 3.**
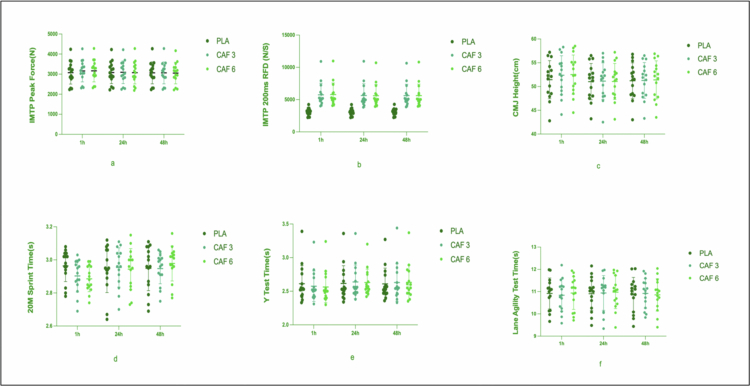
Time-course distribution of performance outcomes. Individual data points and group distributions are shown for IMTP peak force, IMTP 200-ms RFD, CMJ height, 20-m sprint time, Y-shaped reactive agility test time, and Lane Agility Test time under PLA, CAF3, and CAF6 conditions at 1 h, 24 h, and 48 h post-RP.

**Figure 4.**
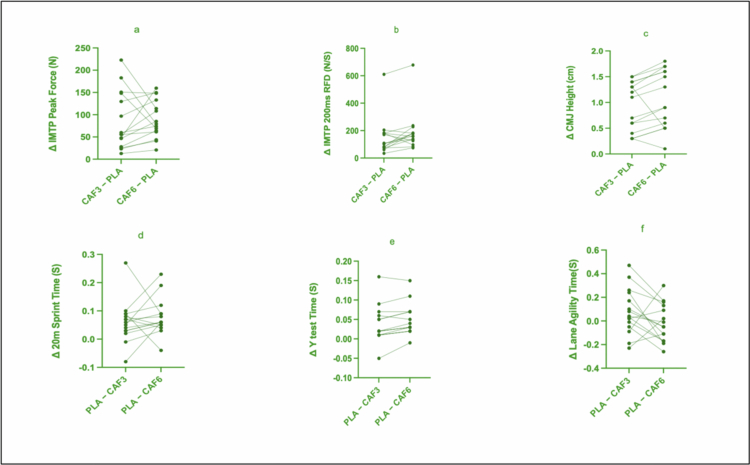
Individual responses to caffeine gum at 1 h post-RP relative to placebo. Individual paired changes are shown for CAF3–PLA and CAF6–PLA comparisons in IMTP peak force, IMTP 200-ms RFD, CMJ height, 20-m sprint time, Y-shaped reactive agility test time, and Lane Agility Test time.

Time-course distributions and individual 1 h responses relative to PLA are shown in Figures 3 and 4, respectively. Descriptive statistics for all performance outcomes are presented in Table 2. Overall, CAF3 and CAF6 showed more favourable performance values than PLA at 1 h for IMTP peak force, 200-ms RFD, CMJ height, 20-m sprint time, and Y-shaped reactive agility test time, whereas between-condition differences were less apparent at 24 h and 48 h. Lane Agility Test performance showed relatively similar values across conditions and time points.

**Table 2.** Descriptive statistics for performance outcomes.

Test	Time	PLA	CAF3	CAF6
Peak Force	1 h	3072.61 ± 566.41	3158.38 ± 544.75	3162.70 ± 549.23
	24 h	3081.47 ± 553.96	3092.10 ± 563.59	3074.02 ± 561.13
	48 h	3063.00 ± 545.73	3062.60 ± 542.91	3053.18 ± 536.24
RFD (N/s)	1 h	5596.94 ± 1690.54	5747.24 ± 1779.99	5786.07 ± 1800.12
	24 h	5575.97 ± 1675.94	5635.17 ± 1823.98	5605.63 ± 1756.50
	48 h	5607.86 ± 1752.99	5623.20 ± 1763.16	5624.70 ± 1783.85
CMJ Height (cm)	1 h	51.44 ± 3.99	52.37 ± 4.10	52.50 ± 4.12
	24 h	51.03 ± 3.81	51.13 ± 3.85	51.06 ± 3.81
	48 h	51.21 ± 3.77	51.28 ± 3.76	51.39 ± 4.02
20-m sprint (s)	1 h	2.96 ± 0.09	2.90 ± 0.10	2.89 ± 0.08
	24 h	2.94 ± 0.14	2.95 ± 0.12	2.94 ± 0.12
	48 h	2.95 ± 0.13	2.95 ± 0.09	2.98 ± 0.11
Y test (s)	1 h	2.61 ± 0.27	2.57 ± 0.23	2.56 ± 0.24
	24 h	2.62 ± 0.26	2.64 ± 0.25	2.63 ± 0.20
	48 h	2.61 ± 0.23	2.63 ± 0.28	2.63 ± 0.26
Lane Agility Test (s)	1 h	10.92 ± 0.69	10.84 ± 0.71	10.94 ± 0.74
	24 h	10.91 ± 0.75	10.94 ± 0.78	10.97 ± 0.74
	48 h	10.87 ± 0.77	10.92 ± 0.66	10.85 ± 0.73

### 3.1. IMTP peak force

Linear mixed-effects model results are shown in Table 3. The model revealed a significant dose × time interaction for peak force (F = 5.553, *p* < 0.001), whereas period and sequence effects were not significant. Bonferroni-adjusted pairwise comparisons showed that, at 1 h, peak force was significantly higher in CAF3 than PLA (*p* < 0.001, Hedges’ g = 1.275, SE = 18.310) and in CAF6 than PLA (*p* < 0.001, Hedges’ g = 2.025, SE = 18.310), with no significant difference between CAF3 and CAF6. No significant between-dose differences were observed at 24 h or 48 h.

**Table 3.** Linear mixed-effects model results and standardised effect sizes for IMTP peak force.

Factors	F	*p*-value	CAF3 vs PLA at 1 h	CAF6 vs PLA at 1 h
Dose	4.996	0.008		
Time	23.983	<0.001		
Dose × time	5.553	<0.001		
Period	0.449	0.639		
Sequence	0.927	0.510		
Hedges’ g (95% CI)			1.275(0.584,1.942)	2.025(1.123,2.904)

Note: The F and p values are from the linear mixed-effects model. Hedges’ g indicates effect sizes for 1 h CAF–PLA comparisons.

#### 3.1.1. IMTP 200-ms RFD

Linear mixed-effects model results are shown in Table 4. The model revealed a significant dose × time interaction for RFD (F = 6.919, *p* < 0.001), whereas period and sequence effects were not significant. Bonferroni-adjusted pairwise comparisons showed that, at 1 h, RFD was significantly higher in CAF3 than PLA (*p* < 0.001, Hedges’ g = 1.061, SE = 27.014) and in CAF6 than PLA (*p* < 0.001, Hedges’ g = 1.284, SE = 27.014), with no significant difference between CAF3 and CAF6. No significant between-dose differences were observed at 24 h or 48 h.

**Table 4.** Linear mixed-effects model results and standardised effect sizes for 200-ms RFD.

Factors	F	*p*-value	CAF3 vs PLA at 1 h	CAF6 vs PLA at 1 h
Dose	16.215	<0.001		
Time	26.707	<0.001		
Dose × time	6.919	<0.001		
Period	2.804	0.065		
Sequence	0.950	0.495		
Hedges’ g (95% CI)			1.061(0.422,1.677)	1.284(0.590,1.953)

**Note**: The F and p values are from the linear mixed-effects model. Hedges’ g indicates effect sizes for 1 h CAF–PLA comparisons.

### 3.2. CMJ height

Linear mixed-effects model results are shown in Table 5. The model revealed a significant dose × time interaction for CMJ height (F = 9.968, *p* < 0.001). A significant sequence effect was also detected (F = 5.536, *p* = 0.013), whereas the period effect was not significant. Therefore, pairwise comparisons were interpreted based on the adjusted LMM. Bonferroni-adjusted comparisons showed that, at 1 h, CMJ height was significantly higher in CAF3 than PLA (*p* < 0.001, Hedges’ g = 1.883, SE = 0.137) and in CAF6 than PLA (*p* < 0.001, Hedges’ g = 1.828, SE = 0.137), with no significant difference between CAF3 and CAF6. No significant between-dose differences were observed at 24 h or 48 h.

**Table 5.** Linear mixed-effects model results and standardised effect sizes for CMJ Height.

Factors	F	*p*-value	CAF3 vs PLA at 1 h	CAF6 vs PLA at 1 h
Dose	16.947	<0.001		
Time	94.356	< 0.001		
Dose × time	9.968	< 0.001		
Period	1.576	0.211		
Sequence	5.536	0.013		
Hedges’ g (95% CI)			1.883(1.024,2.720)	1.828(0.985,2.648)

Note: The F and p values are from the linear mixed-effects model. Hedges’ g indicates effect sizes for 1 h CAF–PLA comparisons.

### 3.3. 20-m sprint

Linear mixed-effects model results are shown in Table 6. The model revealed a significant dose × time interaction for 20-m sprint time (F = 3.642, *p* = 0.008). The sequence effect was not significant, but a significant period effect was observed (F = 3.780, *p* = 0.026), indicating that sprint performance may have been partially influenced by experimental period or testing order; therefore, pairwise comparisons were interpreted based on the LMM adjusted for period and sequence. Bonferroni-adjusted comparisons showed that, at 1 h, 20-m sprint time was significantly shorter in CAF3 than PLA (*p* = 0.022, Hedges’ g = 0.799, SE = 0.022) and in CAF6 than PLA (*p* = 0.001, Hedges’ g = 1.177, SE = 0.022), with no significant difference between CAF3 and CAF6. No significant between-dose differences were observed at 24 h or 48 h.

**Table 6.** Linear mixed-effects model results and standardised effect sizes for 20-m sprint.

Factors	F	*p*-value	CAF3 vs PLA at 1 h	CAF6 vs PLA at 1 h
Dose	1.188	0.309		
Time	5.686	0.004		
Dose × time	3.642	0.008		
Period	3.780	0.026		
Sequence	0.700	0.637		
Hedges’ g (95% CI)			0.799(0.215,1.361)	1.177(0.510,1.820)

Note: The F and p values are from the linear mixed-effects model. Hedges’ g indicates effect sizes for 1 h CAF–PLA comparisons.

### 3.4. Y test

Linear mixed-effects model results are shown in Table 7. The model revealed a significant dose × time interaction for Y-shaped reactive agility test time (F = 7.497, *p* < 0.001). Period and sequence effects were not significant, suggesting that experimental period and administration sequence did not significantly influence this outcome. Bonferroni-adjusted comparisons showed that, at 1 h, Y-shaped reactive agility test time was significantly shorter in CAF3 than PLA (*p* = 0.003, Hedges’ g = 0.752, SE = 0.011) and in CAF6 than PLA (*p* < 0.001, Hedges’ g = 1.174, SE = 0.011), with no significant difference between CAF3 and CAF6. No significant between-dose differences were observed at 24 h or 48 h.

**Table 7.** Linear mixed-effects model results and standardised effect sizes for Y-shaped reactive agility test time.

Factors	F	*p*-value	CAF3 vs PLA at 1 h	CAF6 vs PLA at 1 h
Dose	0.474	0.624		
Time	31.036	< 0.001		
Dose × time	7.497	< 0.001		
Period	0.365	0.695		
Sequence	0.338	0.877		
Hedges’ g (95% CI)			0.752(0.177,1.306)	1.174(0.508,1.816)

Note: The F and p values are from the linear mixed-effects model. Hedges’ g indicates effect sizes for 1 h CAF–PLA comparisons.

### 3.5. Lane agility test

Linear mixed-effects model results are shown in Table 8. No significant dose × time interaction was observed for Lane Agility Test time. In addition, neither the sequence effect nor the period effect was significant, suggesting that dose condition, time point, experimental period, and administration sequence did not significantly influence Lane Agility Test performance.

**Table 8.** Linear mixed-effects model results for Lane Agility Test.

Factors	F	*p*-value
Dose	0.243	0.785
Time	1.203	0.304
Dose × time	0.956	0.435
Period	0.112	0.894
Sequence	0.260	0.924

### 3.6. Barbell MV during RP

**Figure 5.**
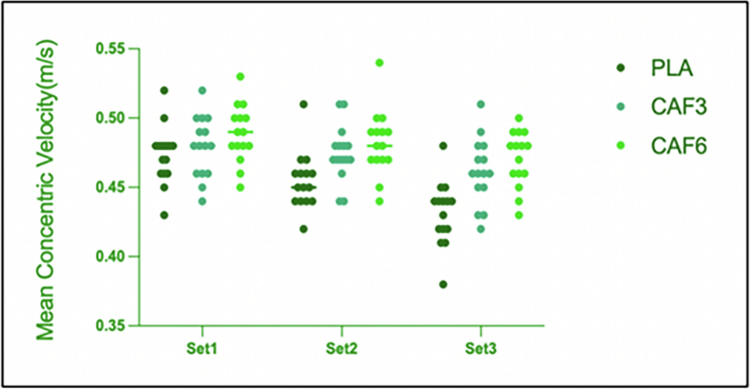
MV during the RP back squat across sets under the PLACAF3and CAF6 conditions. Individual data points are shown for each set of the standardised 3 × 3 back squat protocol at 85% 1RM.

The linear mixed-effects model showed a significant dose × set interaction for MV (F = 6.443, *p* < 0.001), whereas the sequence and period effects were not significant. Bonferroni-adjusted pairwise comparisons showed that, in Set 1, only CAF6 was significantly higher than PLA (*p* < 0.001). In Set 2, both CAF3 and CAF6 were significantly higher than PLA (both *p* < 0.001). Similarly, in Set 3, both CAF3 and CAF6 were significantly higher than PLA (both *p* < 0.001). No significant differences were observed between CAF3 and CAF6 within any set (Figure 5).

## 4. Discussion

This study investigated the acute effects and subsequent time course of different doses of caffeinated chewing gum on neuromuscular performance in male basketball players under a standardised RP condition. The results showed that, except for the Lane Agility Test, both CAF3+RP and CAF6+RP produced superior performance outcomes at 1 h post-ingestion compared with PLA+RP, with no significant differences between the two caffeine doses. However, no significant differences among the three intervention conditions were observed at 24 h or 48 h for any performance outcome. In addition, barbell MV during the RP back squat was higher under the CAF conditions than under PLA, suggesting that caffeinated chewing gum ingestion may be associated with greater RP training output and improved same-day performance.

Under the standardised RP condition used in the present study, caffeinated chewing gum ingestion may be associated with greater RP training output and improved same-day performance. Abdioglu et al [31]. reported that 100 mg of caffeinated chewing gum significantly reduced perceived exertion (RPE) during tennis-specific tasks. Similarly, Mor et al [32]. found that CAF6 significantly enhanced exercise performance in male soccer players, suggesting that caffeine, as an adenosine receptor antagonist, may block A1/A2A receptors, reduce endogenous inhibition, increase neurotransmitter release, and enhance Na⁺/K⁺-ATPase activity in skeletal muscle, thereby facilitating excitation–contraction coupling. These findings suggest that caffeine ingestion may increase arousal and reduce perceived fatigue, allowing participants to maintain higher movement velocity during RP. In addition, high-intensity resistance exercise (>80% 1RM) itself promotes the recruitment of high-threshold motor units [33]. Therefore, the greater RP training output observed under CAF conditions may have contributed to the performance advantages observed at 1 h. However, these explanations are mainly inferential and are based on previous studies and the present performance outcomes. The present study did not directly measure the relevant physiological or neuromuscular mechanisms and did not include a caffeine-only condition or a no-RP control group. Therefore, the current findings only indicate that, within a standardised RP context, caffeine ingestion was associated with higher barbell MV and superior 1 h performance, but they do not provide direct evidence of a clear synergistic effect between caffeine and RP. Notably, Amor et al [34]. reported that the effects of caffeine may partly depend on the route of administration. Accordingly, the acute performance benefits observed at 1 h in the present study may reflect the combined effect of caffeinated chewing gum under natural chewing and swallowing conditions. However, because salivary or plasma caffeine concentrations were not measured, we cannot confirm whether the performance assessments coincided with peak systemic caffeine concentrations; therefore, this interpretation should be considered with caution.

It should be emphasised that the present study did not observe any additional between-condition advantage of CAF over PLA at 24 h or 48 h under the standardised RP condition. However, because all conditions included RP and the study did not include pre-intervention baseline assessments, a caffeine-only condition, or a no-RP control group, the presence of RP-related delayed enhancement at 24 h or 48 h cannot be directly determined from the present design. Likewise, the current findings cannot establish whether performance in any condition remained above participants’ initial baseline levels at 24 h or 48 h, or whether RP itself induced delayed subsequent effects. Therefore, a more appropriate interpretation is that, within the standardised RP context, different doses of CAF did not produce additional between-condition advantages over PLA at 24 h or 48 h. Given that barbell MV during RP was higher under CAF conditions, whereas no additional between-condition advantages were observed at 24 h or 48 h, one possible explanation is that the increase in MV under CAF conditions primarily reflected an acute enhancement of RP training output [35]. This acute modulation may have been sufficient to improve same-day training output and immediate performance, but not sufficient to further amplify between-condition differences at later time points under the present RP protocol. Nevertheless, because neuromuscular mechanisms were not directly measured and a no-RP control group was not included, this explanation remains speculative.

The present study found that, under a standardised RP context, both CAF3+RP and CAF6+RP produced superior outcomes at 1 h compared with PLA+RP, whereas no significant differences were observed between the two caffeine doses. Previous research [36] reported that acute CAF6 ingestion significantly improved repeated-sprint performance in female soccer players, suggesting that the potential advantage of a higher caffeine dose may be more evident during repeated or prolonged high-intensity exercise. In contrast, the present testing design involved relatively limited fatigue accumulation, which may have reduced the likelihood of detecting dose-dependent differences. Moreover, current evidence indicates that increasing caffeine dose does not necessarily produce additional or more comprehensive performance benefits. Acar et al. [37] found that CAF6 improved sprint swimming performance in female swimmers but did not significantly enhance CMJ, reaction time, or other performance outcomes. This suggests that caffeine’s effects may depend not only on dose, but also on task characteristics, participant profile, and testing context. Therefore, the present findings indicate only that CAF3 and CAF6 elicited similar performance responses in the current sample, outcome measures, and standardised RP condition. These results should not be interpreted as evidence that CAF3 is sufficient for all practical scenarios, and the applied value of different caffeine doses requires further validation in larger samples and more competition-specific sport settings.

In the present study, no significant improvement was observed in the Lane Agility Test. This finding does not necessarily indicate that CAF combined with RP was entirely ineffective for this task, but may partly be related to the relatively high coefficient of variation (CV) of the test. Previous research [38] reported a CV of 7.3% for the Lane Agility Test in youth basketball players, with the typical error exceeding the smallest worthwhile change. This suggests that the test may have limited sensitivity for detecting small but practically meaningful performance changes. Moreover, greater test variability may reduce the statistical sensitivity to detect small effects, thereby potentially masking subtle performance changes [39]. Therefore, the Lane Agility Test results in the present study should be interpreted as indicating that, under the current sample size, testing conditions, and RP protocol, CAF3+RP and CAF6+RP did not produce a detectable additional performance advantage compared with PLA+RP, rather than being attributed to a single underlying mechanism.

## 5. Limitations

Several limitations should be acknowledged. First, the sample size was small and included only 15 healthy male basketball players with low habitual caffeine intake, limiting the generalisability of the findings to female athletes, elite players, different age groups, and individuals with higher habitual caffeine consumption. Second, although dietary intake and caffeine habits were recorded, these data were self-reported and not objectively verified. Salivary or plasma caffeine concentrations and caffeine metabolism-related genotypes, such as CYP1A2, were also not assessed, preventing confirmation of whether the 1-h performance assessments coincided with peak systemic caffeine concentrations or further explanation of inter-individual response variability. In addition, because caffeine gum was chewed for 15 min under natural chewing and saliva-swallowing conditions, and chewing frequency was not standardised or monitored, individual differences in mastication rate, buccal absorption, and gastrointestinal absorption may have affected caffeine release from the gum matrix and the actual caffeine dose reaching systemic circulation. Third, the study did not include trial-day pre-intervention baselines, a caffeine-only condition, or a no-RP control group. Therefore, daily readiness, performance relative to baseline at 24 h and 48 h, and the independent delayed effects of RP could not be directly determined. Fourth, the RP protocol used back squats, which mainly involve sagittal-plane loading, whereas basketball requires more complex multiplanar movements; thus, ecological validity remains limited. Finally, although 5-day washout period was used and period and sequence effects were included in the LMM, learning effects, test familiarisation, or residual neuromuscular fatigue cannot be completely excluded. Future studies should use larger and more diverse samples, include objective physiological measures, trial-day baselines, caffeine-only and no-RP controls, and more basketball-specific RP protocols.

## 6. Conclusion

The present findings indicate that, within a standardised RP context, CAF3 and CAF6 gum ingestion was associated with superior neuromuscular performance at 1 h compared with PLA+RP. However, no additional between-condition advantages were observed for CAF3+RP or CAF6+RP over PLA+RP at 24 h or 48 h, and no significant differences were detected between the two caffeine doses. Because the present study did not include pre-intervention baseline testing, a caffeine-only condition, or a no-RP control condition, these findings should be interpreted as additional between-condition effects of caffeine relative to placebo under a standardised RP protocol, rather than direct evidence of the independent effects of caffeine, the independent effects of RP, or a caffeine × RP interaction. Similarly, the present design cannot determine whether RP induced delayed performance enhancement at 24 h or 48 h. Therefore, the absence of additional CAF-related advantages over PLA at the later time points should not be interpreted as evidence that caffeine failed to prolong or maintain RP-induced residual benefits.
